# User-Perceived Capacity: Theory, Computation, and Achievable Policies

**DOI:** 10.3390/e26110914

**Published:** 2024-10-28

**Authors:** Yuanrui Liu, Xiaoyu Zhao, Wei Chen

**Affiliations:** 1 Department of Electronic Engineering, State Key Laboratory of Space Network and Communications, Beijing National Research Center for Information Science and Technology, Tsinghua University, Beijing 100084, China; liuyr19@mails.tsinghua.edu.cn (Y.L.); wchen@tsinghua.edu.cn (W.C.); 2 School of Cyber Science and Engineering, Southeast University, Nanjing 211189, China

**Keywords:** user-perceived capacity, ultra-reliable and low-latency communications, real-time wireless communication, hard delay constraint, cross-layer scheduling, finite horizon Markov decision processes

## Abstract

User-perceived throughput is a novel performance metric attracting a considerable amount of recent attention because it characterizes the quality of the experience in mobile multimedia services. For instance, it gives a data rate of video streaming with which a user will not experience any lag or outage in watching video clips. However, its performance limit remains open. In this paper, we are interested in the achievable upper bound of user-perceived throughput, also referred to as the user-perceived capacity, and how to achieve it in typical wireless channels. We find that the user-perceived capacity is quite limited or even zero with channel state information at the receiver (CSIR) only. When both CSIR and channel state information at the transmitter (CSIT) are available, the user-perceived throughput can be substantially improved by power or even rate adaptation. A constrained Markov decision process (CMDP)-based approach is conceived to compute the user-perceived capacity with joint power–rate adaptation. It is rigorously shown that the optimal policy obeys a threshold-based rule with time, backlog, and channel gain thresholds. With power adaptation only, the user-perceived capacity is equal to the hard-delay-constrained capacity in our previous work and achieved by joint diversity and channel inversion.

## 1. Introduction

Future 6G communication aims to provide a revolutionary user experience [1,2], with real-time communication holding high expectations in achieving the goals of future mobile communications. On the other hand, the user-perceived capacity is a novel performance metric that has attracted considerable recent attention because it characterizes the quality of experience in mobile multimedia services [3]. In this paper, our objective is to improve the user-perceived capacity to meet the demands of real-time communication in future wireless communications.

Real-time communication is essential for applications where latency is a critical factor. It ensures that the time taken for data to travel from the transmitter to the receiver is within acceptable limits, enabling seamless and interactive experiences. Study [4] indicated that communication in real-time systems must be predictable, and a scheme was proposed for providing predictable inter-process communication in real-time systems with a point-to-point interconnection network. Study [5] focused on the time sensitive networks, analyzed the key functional parameters affecting the deterministic behavior in real-time communication protocols and, based on the configuration of these parameters, derived the constraints required for computing schedules to ensure the deterministic end-to-end delay of critical communication flows. In [6], the authors concentrated on real-time communication for industrial automation environments, aiming to meet the demands of real-time communication in a scenario where the number of networked devices and computational capabilities are expected to significantly increase. The authors of [7] focused on wireless sensor networks, summarizing and employing various methods based on both hard real-time and soft real-time models. Furthermore, they provided scheduling policies to ensure the requirements of real-time communication. In particular, hard real-time communication implies that missing the deadline can lead to a system-wide failure, necessitating the satisfaction of end-to-end latency constraints within the deadline. On the other hand, soft real-time communication aims to reduce the transmission of packets that exceed the deadline, that is, to minimize the probability of exceeding the deadline. In this paper, we focus on hard latency constraints. This is due to hard latency constraints playing an extremely important role in the ultra-reliable and low-latency communications (URLLC) scenario, ensuring timely and reliable data delivery [8]. Many studies have primarily targeted hard latency constraints, such as [9,10,11,12,13,14,15,16,17]. In the literature, the authors of [10] considered a communication network under hard real-time constraints, wherein the source node transmits information to the destination node via a directed acyclic graph subject to hard latency constraints. In [9], the authors demonstrated that hard latency constraints can be provided using frequency or spatial diversity and time domain power adaptation, with the condition that diversity gains are at least two. Study [9] also revealed the underlying relationships between the required average power, throughput, and outage probability. The authors of [12] presented a novel power control scheme enhancing the link layer performance in delay-sensitive communications over fading channels. This scheme is particularly adept at managing joint queue length awareness. In [13], the authors introduced an optimal dynamic coding algorithm, along with its low-complexity approximations, designed for deadline-constrained traffic. Additionally, a joint coding and rate control policy proposed by [13] outperformed in time-varying channels, making it particularly beneficial for multimedia streaming and interactive real-time applications. This proposed policy was demonstrated to offer superior performance and stability. However, there is also a considerable amount of research that concentrates on soft real-time communications, specifically addressing the issue of delay violation probabilities over wireless channels [18]. Refs. [19,20] contributed to the field by formulating a CMDP to minimize the queue-length-bound violation probability, utilizing linear programming to obtain the optimal probabilistic policy, balancing violation probability with transmission power. The authors of [21] proposed a framework for cross-layer optimization to ensure the required quality-of-service with finite transmit power, considering the transmission error probability, queueing delay violation probability, and packet dropping probability. Liu et al. proposed a URLLC-centric task offloading and resource allocation framework, incorporating the statistical characteristics’ extreme queue length events [22]. In [23], the authors focused on reducing packet delay violation probabilities within periodic traffic models and, by utilizing Markov decision processes and nonlinear Knapsack Problems, presented an asymptotically optimal policy.

In the context of real-time communication, wireless networks are responsible for transmitting substantial amounts of data in a specified time frame, with an increasing emphasis on ensuring high service quality. A significant portion of data has strict latency requirements, which arise from various application scenarios involving real-time communication, such as autonomous driving, health monitoring, and industrial automation. To meet these latency requirements, data transmissions must be completed within a given deadline constraint. Consequently, Zafer and Modiano proposed a BT problem in [24], addressing the challenge of transmitting B units of data within a deadline T. They employed continuous-time stochastic control theory to derive the optimal policy for the BT problem. In addition, Berry et al. provided an overview of research related to packet delay and energy consumption in [25], including the BT problem and delay-constrained issues. For discrete-time point-to-point communication systems, refs. [26,27] investigated the optimal data transmission for BT problems under constraints such as random data arrival and Quality of Service (QoS), without considering time-varying channels. To specifically address the impact of time-varying channel conditions on the BT problem, several studies [11,28,29] extended the BT problem from static channels to fading channels. Furthermore, studies [24,30,31,32] have considered the BT problem in continuous-time systems, exploring the optimal data transmission policies in the presence of time-varying channels. In addition to single-user scenarios, the academic community has also conducted research on multi-user scenarios. For instance, the authors of [33] investigated the BT problem in a time-shared multi-user system by considering random arrivals and time-varying channels for multiple users. Furthermore, study [34] extended the BT problem to multi-access scenarios and examined power minimization scheduling with deadline constraints in a multi-access channel environment. Moreover, under the framework of the BT problem, studies such as [35,36,37,38,39] further explored deadline-constrained communications in scenarios such as multi-hop networks, computation offloading, lossy networks, and multi-robot task allocation, providing optimal scheduling policies.

Cross-layer design is widely recognized as a crucial methodology for ensuring real-time performance for delay-constrained services. Notably, Collins and Cruz pioneered the integration of network and physical layer designs to optimize low-latency wireless transmissions, resulting in the formulation of a cross-layer scheduling framework [40]. After that, there has been an increasing focus on interactions across different layers [41,42,43], leading to the proposal of various approaches for cross-layer scheduling in diverse application scenarios. Specifically, Lyapunov optimization and Markov decision processes are commonly used in cross-layer designs to enhance system throughput and reduce latency. Inspired by the existing literature on cross-layer scheduling approaches [40,41,42,43], researchers utilized Lyapunov optimization to describe data arrivals at the network layer and channel state information at the physical layer, thereby exploring power-constrained cross-layer scheduling for low-latency wireless communications. In [44], the authors introduced online dynamic control algorithms using Lyapunov optimization theory for multi-hop networks. In [45], Neely provided a systematic summary of Lyapunov optimization theory, focusing primarily on communication and queuing systems. Building upon this, refs. [46,47,48] demonstrated the throughput optimality of the Lyapunov drift method under the maximum weight rule, the logarithmic rule, and the exponential rule in multi-user scenarios over time-varying wireless channels. Subsequently, the Lyapunov drift method has been widely applied in packet switching [49,50,51], queuing networks [52], wireless communication [53,54], and satellite communication systems [55]. When the system exhibits Markovian properties, a Markov decision process (MDP) can be employed to achieve a more rigorous analysis and optimization for metrics such as delay and power in wireless communication systems [56,57]. Berry and Gallager conducted an analysis on the average delay and power for a single-user scenario over block-fading channels in discrete-time systems [58]. Building upon this, ref. [59] studied the trade-off among the average power, delay, and the packet drop ratio in a single-user link over fading channels. It provided deterministic policies for unconstrained problems and probabilistic policies for constrained problems using value iteration and linear programming, respectively. Refs. [60,61] further investigated the trade-off between delay and power for random arrivals in AWGN and fading channels based on the constrained MDP (CMDP) framework. Specifically, the authors showed structural properties of the optimal scheduling policy, such as a threshold-based structure and piecewise linearity, and consequently developed an efficient algorithm to search for the optimal delay-power tradeoff. As an extension of [60,61], ref. [62] considered a Markovian arrival process, and showed similar threshold and piecewise linear characteristics for the optimal scheduling policy. Under a CMDP framework with cross-layer designs, refs. [63,63] presented the optimal delay-power trade-off for two-user systems over AWGN and block-fading channels, respectively.

In the aforementioned references, the studies from [40,41,42,43,44,45,46,47,48,49,50,51,52,53,54,55,56,57,58,59,60,61,62,63,64] focus on the research of cross-layer design and do not conduct specific studies on real-time communication. Although the references from [4,5,6,7,8,9,10,11,12,13,14,15,16,17,18,19,20,21,22,23] primarily concentrate on summarizing and designing the framework of real-time communication, they do not specifically address the issue of transmitting B units of data within a deadline T. The studies from [24,25,26,27,28,29,30,31,32,33,34,35,36,37,38,39], while focusing on the BT problem, do not take into account the user-perceived capacity, that is, they do not consider how to meet the requirement for the continuous reading of the user. Based on this, this paper focuses on real-time communication, utilizing the framework of cross-layer design to improve the user-perceived capacity under the constraint of average power.

This paper primarily investigates single-user communication systems over wireless fading channels. The objective is to maximize the user-perceived capacity under the constraint of average power. Specifically, we consider periodic data arrivals, with B packets of data arriving at the beginning of each period. The user reads the received data at a certain rate at the receiver. To satisfy the user-perceived capacity, we need to transmit B units of data within a deadline T. In particular, the main contributions of the paper are as follows.

- We model the user-perceived capacity-oriented system as a two-dimensional Markov chain with the queue length and time index as the system state. Based on this, we rigorously analyze the power consumption and show conditions for ensuring a target user-perceived capacity.
- Under this Markov chain, we formulate a dual problem of maximizing the user-perceived capacity over fading channels, i.e., minimizing the average power while achieving a constraint on the user-perceived capacity. To address the non-convexity of this dual problem, we convert it into a linear programming problem, equivalently, via a variable combination method.
- Next, we obtain an optimal cross-layer scheduling policy by solving the derived linear programming problem. Subsequently, a binary search method is developed to acquire the maximum user-perceived capacity under an arbitrary average power constraint.
- Furthermore, we represent the single user link as a finite horizon Markov decision process, and demonstrate a series of threshold structures of the optimal policy in terms of queue length, time-slot, and channel state.

The rest of this paper is organized as follows. Section 2 initially provides a detailed definition of user-perceived capacity. Section 3 illustrates the system model. The BT problem with the cross-layer approach is investigated in Section 4. In Section 5, we prove the structure of the optimal policy. Numerical results and concluding remarks are presented in Section 6 and Section 7, respectively.

## 2. User-Perceived Capacity

We define *C* and R(t) as the user-perceived capacity and transmission rate, respectively. For the time duration [0,T], the user-perceived capacity *C* satisfies

(1) $$Ct\leq \int_{0}^{t}R\left(\tau \right)d\tau ,\;\;w.p.\;1,\;\forall t\in \left[0,T\right].$$

In discrete-time systems, the integral transforms into the form of a summation $\sum_{t=0}^{T}R\left(t\right)$. Specifically, the user-perceived capacity refers to the capacity that enables users to avoid any interruptions during reading. Then, we obtain the following three propositions.

**Proposition** **1.** *In the AWGN channel, the user-perceived capacity C is equal to*

(2) $$C=W\log_{2}\left(1+\gamma \right)\;\mathrm{bit}/s,$$

*where W and γ represent the bandwidth and the signal-to-noise ratio at the receiver, respectively.*

**Proposition** **2.** *In the fading channel with the channel state information at the receiver, each positive constant c>0 satisfies*

(3) $$\mathrm{Pr}\left\{ct>\int_{0}^{t}R\left(t\right)dt\right\}>0,\;\forall t\in \left[0,T\right],$$

*due to the unbounded power under deep fading. As such, the user-perceived capacity C is equal to zero.*

**Proposition** **3** (Our previous work [9])**.** *In the fading channel with the channel state information at the transmitter and the receiver, the user-perceived capacity is equal to the hard-delay-constrained capacity with power adaptation only, i.e.,*

(4a) $$\begin{array}{cc} \; & C=W\log_{2}\left(1+\gamma \right)\;\mathrm{bit}/s, \end{array}$$

(4b) $$\begin{array}{cc} \; & P=E_{h}\left\{\frac{\gamma \sigma^{2}}{h^{2}}\right\}, \end{array}$$

*where P denotes the expectation of transmission power over the random channel gain h. Meanwhile, $\sigma^{2}$ denotes the noise variance. (The condition for Proposition 3 to hold is that the power P is bounded as stated in Equation (4b). The reader may have observed that Equation (4b) does not hold under Rayleigh fading channels. In response to this case, we have demonstrated in our paper [9] that when the frequency or spatial diversity order exceeds two, Equation (4b) can be satisfied.).*

In this paper, leveraging a cross-layer approach, we achieve the user-perceived capacity with the rate adaptation. Our proposed policy yields a user-perceived capacity that is inevitably superior to that presented in Proposition 3.

## 3. System Model

In this paper, we consider a data transmission from a base station to a user. When a user request is sent to the base station, the base station shall send an amount of data to the user, supporting a target experience rate within a deadline, i.e., meeting the user-perceived capacity. Specifically, a target rate illustrates how fast the user reads the transmitted data at the receiver. Due to the randomness of the wireless channel, a careful channel resource arrangement shall be acquired to avoid any interruption for reading the data.

### 3.1. Queueing Model

**Figure 1:**
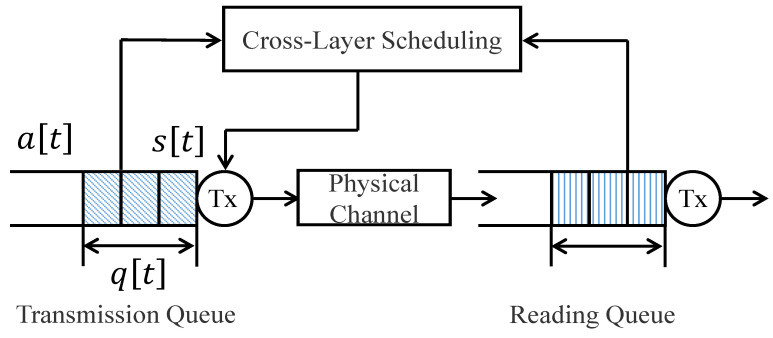
System model.

As shown in Figure 1, the time is divided into time-slots, each of which spans τ s and is indexed by t=0,1,2,…. We define a[t] as the number of packets that arrive at the base station in the beginning of time-slot *t*. Meanwhile, we suppose that the data packet arrival follows a periodical process with the arrival period of *T* time-slots and the number of arriving packets set as *B*.

As such, a[t] evolves as

(5) $$a\left[t\right]=\left\{\begin{array}{cc} B & \mathrm{if}\;t\;\mathrm{mod}\;T=0,\\ 0 & \mathrm{if}\;t\;\mathrm{mod}\;T\neq 0. \end{array}\right.$$

According to the classic BT problem proposed in [25], these *B* data packets that arrive periodically must be transmitted within *T* time-slots.

Let s[t] represent the transmission rate, which is the number of packets transmitted in time-slot *t*. Owing to the constrained throughput at the base station, we set an upper bound of s[t] by *S*. This way, the feasible region of s[t] is defined as S={0,1,2,…,S}.

Meanwhile, a buffer of size *Q* is utilized to store remaining packets at the base station. Let q[t] denote the queue length, i.e., the number of packets backlogged in the buffer, in the *t*-th slot. As such, we have q[t]∈Q={0,1,2,…,Q}. The queue state q[t] evolves as

(6) $$q\left[t+1\right]=\min\{{\left(q\left[t\right]-s\left[t\right]\right)}^{+}+a\left[t+1\right],Q\},$$

where ${\left(x\right)}^{+}=\max\left\{x,0\right\}$.

### 3.2. Physical Layer Model

In the physical layer, we represent the signal transmitted during the *t*-th time-slot as X[t], and the corresponding signal received at the receiver as Y[t]. The channel gain is denoted as h[t]. We express the received signal as follows.

(7) Y[t]=h[t]X[t]+Z[t],

where Z[t] represents an additive white Gaussian noise process.

Note that a quantized channel state is typically used to determine modulation and coding schemes’ real-world systems due to the limited resources for channel estimation. As such, we employ a set of thresholds for the quantization of the channel gain h[y], namely, $h_{0}^{th}=0<h_{1}^{th}<\cdots <h_{L}^{th}=+\infty$. Specifically, we set h[t] as $h_{l}=\frac{1}{2}\left(h_{l-1}^{th}+h_{l}^{th}\right)$ for l=1,…,L, if the actual channel gain falls within the interval of $\left[h_{l-1}^{th},h_{l}^{th}\right)$. Consequently, we can model h[t] as an *L*-state block-fading channel, with $H=\{h_{1},h_{2},\ldots ,h_{L}\}$ representing the feasible region of channel states, characterized by $0<h_{1}<h_{2}<\cdots <h_{L}<+\infty$. Furthermore, we define the probability of $h\left[t\right]=h_{l}$ as follows.

(8) $$\eta_{l}=\int_{h_{l-1}^{th}}^{h_{l}^{th}}p(x)dx\;\mathrm{for}\;l=1,2,\ldots ,L,$$

where p(x) is the probability density function of the channel gain that is conducted from the real-world wireless environment. Additionally, h[t] is independently and identically distributed (i.i.d.) across time-slots. We assume that the channel state information h[t] is available at the transceiver and receiver at each timeslot. The assumption of the full knowledge of the channel state is widely adopted in the literature and grounded in the CSI measuring and reporting processes stipulated for 5G NR systems in the 3GPP standard [65]. The effect of channel estimation error is also investigated by numerical simulations.

We denote by P[t] the power consumption in the *t*-th time-slot. When the transmission rate s[t]=s and the channel state $h\left[t\right]=h_{l}$, the power consumption P[t]=P(s,l). Without the loss of generality, we assume that P(s,l) is convex with respect to *s* and that it is monotonically increasing. Meanwhile, *S* is the maximum value of transmission rate s[t].

### 3.3. Reading Model

The transmitted packets are stored in a read-only buffer at the user side, and subsequently read under a fixed rate. In the context of the classic BT problem, the reading rate of the user is typically set as $\frac{B}{T}$ packets per time-slot. To avoid any interruption to the reading process at the user, we next develop a continuous reading constraint for the transmission of *B* data packets within an arrival period, i.e., *T* time-slots. To this end, we first denote by m[t] the index of the current time-slot over its corresponding arrival period, which is represented by

(9) m[t]=tmodT.

**Figure 2:**
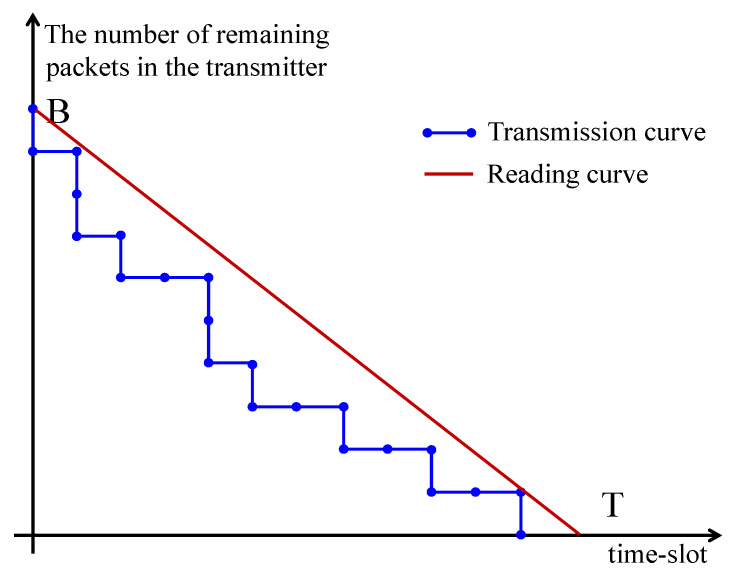
Reading mode. The blue step-change line represents the number of packets remaining as backlog at the base station in each time-slot in a period. The red line represents the number of packets remaining that are unread at the user side.

Here, we have m[t]∈M={0,1,2,…,T−1}. As shown in Figure 2, we can avoid the interruption of the data reading process, as long as the amount of remaining unread data at the user side (red line) is no less than the amount of remaining in-transmitted data at the base station side (blue line). This way, we formulate the continuous reading constraint as

(10) $$\frac{B-q[t]}{m[t]}\geq \frac{B}{T}.$$

To circumvent the influence of the zero point, we introduce

(11) q[t]T+m[t]B≤TB.

Subsequently, we find that optimizing the user-perceived capacity directly, under the constraint of average power, is highly challenging. Therefore, we consider optimizing the dual problem of improving the user-perceived capacity, which minimizes the average power while satisfying the user-perceived capacity. Based on this, we formulate an optimization problem to minimize the long-term average transmission power consumption under the continuous reading constraint in Equation (10), i.e.,

(12a) $$\begin{array}{cc} \min_{\left\{s[t]\in S\right\}} & \lim_{N\to +\infty }\frac{1}{N}\sum_{t=1}^{N}P\left[t\right] \end{array}$$

(12b) $$\begin{array}{cc} s.t.\; & q[t]T+m[t]B\leq TB, \end{array}$$

(12c) $$\begin{array}{cc} \; & q\left[t+1\right]=\min\{{\left(q\left[t\right]-s\left[t\right]\right)}^{+}+a\left[t+1\right],Q\}, \end{array}$$

(12d) $$\begin{array}{cc} \; & m[t]=t\;\;\;\;\mathrm{mod}\;T. \end{array}$$

Before leaving this section, we shall emphasize that the system determines the transmission rate s[t] by jointly considering the current values of the queue state q[t], the channel state h[t], and the time index m[t]. As such, an energy-efficiency scheduling policy is conducted to achieve the user-perceived capacity.

## 4. The Cross-Layer Approach

In this section, we prioritize addressing the dual problem of enhancing the user-perceived capacity, that is, minimizing the average power while meeting the user-perceived capacity requirements. Subsequently, we propose a search algorithm capable of determining the achievable user-perceived capacity under power constraints. Firstly, we delve into a detailed exposition of the scheduling policy, aiming to minimize the average power consumption while ensuring a continuous supply of packets for the user. Specifically, we analytically describe the scheduling policy under a probabilistic method, which is comprised of a conditional probability distribution of s[t] under different values of q[t], h[t], and m[t]. Based on this, we represent the system as a Markov chain with the system state being the pair of the queue length and time index, i.e., (q[t],m[t]). By exploiting the steady-state distribution of the Markov chain and the probabilistic scheduling policy, we re-write problem (12) as a linear programming problem. By solving the derived linear programming problem, we obtain the minimum average power under the continuous reading constraint, as well as the corresponding scheduling policy.

### 4.1. The Probabilistic Scheduling Policy and Markov Chain

We develop a probabilistic scheduling policy based on the queue state, the time index, and the channel state. To this end, we use $f_{q,m,l}^{s}$ to characterize the probabilistic scheduling policy, which is given by

(13) $$f_{q,m,l}^{s}=\mathrm{Pr}\left\{s\left[t\right]=s|q\left[t\right]=q,m\left[t\right]=m,h\left[t\right]=h_{l}\right\}.$$

Moreover, we have

(14) $$\sum_{s\in S}f_{q,m,l}^{s}=1,\;\forall q\in Q,m\in M,h_{l}\in H.\;$$

Given a policy $F=\left\{f_{q,m,l}^{s}\right\}$, we denote by $\pi_{F}$ the steady-state distribution of the Markov chain. Then, we have

(15) $$\pi_{F}=\left[\pi_{F}\left(q,m\right)\right],\;q\in Q,m\in M,$$

where $\pi_{F}\left(q,m\right)$ is the steady-state probability of q[t]=q and m[t]=m. Considering the normalization condition, we have

(16) $$\sum_{q\in Q}\sum_{m\in M}\pi_{F}\left(q,m\right)=1.$$

For each given scheduling policy F, the following lemma presents the transition probability of the Markov chain, defined as

(17) $$\lambda_{\left(q,m\right),\left(\tilde{q},\tilde{m}\right)}=\mathrm{Pr}\left\{\left(q\left[t+1\right],m\left[t+1\right]\right)=\left(\tilde{q},\tilde{m}\right)|\left(q\left[t\right],m\left[t\right]\right)=\left(q,m\right)\right\}.\;$$

**Lemma** **1.** *The transition probability is equal to*

(18) $$\lambda_{\left(q,m\right),\left(\tilde{q},\tilde{m}\right)}=\left\{\begin{array}{cc} \sum_{h_{l}\in H}\sum_{s\in S}\eta_{l}f_{q,m,l}^{s}1\left\{\tilde{q}=q-s\right\}1\left\{\tilde{m}=m+1\right\} & \mathrm{if}\;m<T-1,\\ \;1\left\{\tilde{q}=B\right\}1\left\{\tilde{m}=0\right\} & \mathrm{if}\;m=T-1, \end{array}\right.$$

*where 1{·} is the indicator function.*

**Proof.**  When m<T−1, the transition to the next state does not involve packet arrival. Thus, the queue state transition depends solely on the transmission rate, leading to a change from *q* to q−s with probability $f_{q,m,l}^{s}$. Meanwhile, the time index will inevitably increase by 1. Thus, we have

(19) $$\lambda_{\left(q,m\right),\left(\tilde{q},\tilde{m}\right)}=\sum_{h_{l}\in H}\sum_{s\in S}\eta_{l}f_{q,m,l}^{s}1\left\{\tilde{q}=q-s\right\}1\left\{\tilde{m}=m+1\right\},m<T-1.$$

When m=T−1, the queue state will definitely transition to *B*, and the time index will be reset to zero.    □

Based on Lemma 1, the balance equation is expressed as

(20) $$\sum_{q\in Q}\sum_{m\in M}\lambda_{\left(q,m\right),\left(\tilde{q},\tilde{m}\right)}\pi_{F}\left(q,m\right)=\pi_{F}\left(\tilde{q},\tilde{m}\right).$$

### 4.2. The Power Metric and the Continuous Reading Constraint

We next express the long-term average power consumption in terms of the steady-state distribution $\pi_{F}$ and the scheduling policy F as follows.

**Theorem** **1.** *The long-term average power consumption $P_{ave}=\lim_{N\to +\infty }\frac{1}{N}\sum_{t=1}^{N}P\left[t\right]$ is calculated as*

(21) $$P_{ave}=\sum_{q\in Q}\sum_{m\in M}\sum_{h_{l}\in H}\sum_{s\in S}P\left(s,l\right)\eta_{l}\pi_{F}\left(q,m\right)f_{q,m,l}^{s}.$$

**Proof.**  We calculate the average power consumption from a probability perspective. The probability of the queue state q[t]=q, time index m[t]=m, channel state $h\left[t\right]=h_{l}$, and transmission rate s[t]=s is given by $\eta_{l}\pi_{F}\left(q,m\right)f_{q,m,l}^{s}$, with a power consumption of P(s,l). Subsequently, proving Theorem 1 involves computing the mathematical expectation.    □

The continuous reading constraint in Equation (10) can be transformed into

(22) $$\sum_{q\in Q}\sum_{m\in M}\pi_{F}\left(q,m\right)1\left\{qT+mB>TB\right\}=0.$$

Therefore, we formulate an optimization problem to minimize the average power while satisfying the continuous reading constraint,

(23a) $$\begin{array}{cc} \;\;\;\min_{\left\{\pi_{F}\left(q,m\right),f_{q,m,l}^{s}\right\}}\;\; & \sum_{q\in Q}\sum_{m\in M}\sum_{h_{l}\in H}\sum_{s\in S}P\left(s,l\right)\eta_{l}\pi_{F}\left(q,m\right)f_{q,m,l}^{s} \end{array}$$

(23b) $$\begin{array}{cc} s.t. & \sum_{q\in Q}\sum_{m\in M}\pi_{F}\left(q,m\right)1\left\{qT+mB>TB\right\}=0, \end{array}$$

(23c) $$\begin{array}{cc} \; & \sum_{s\in S}f_{q,m,l}^{s}=1,\forall q\in Q,m\in M,h_{l}\in H, \end{array}$$

(23d) $$\begin{array}{cc} \; & \sum_{q\in Q}\sum_{m\in M}\pi_{F}\left(q,m\right)=1, \end{array}$$

(23e) $$\begin{array}{cc} \; & \sum_{q\in Q}\sum_{m\in M}\lambda_{\left(q,m\right),\left(\tilde{q},\tilde{m}\right)}\pi_{F}\left(q,m\right)=\pi_{F}\left(\tilde{q},\tilde{m}\right),\forall \tilde{q}\in Q,\tilde{m}\in M, \end{array}$$

(23f) $$\begin{array}{cc} \; & \pi_{F}\left(q,m\right)\geq 0,f_{q,m,l}^{s}\geq 0. \end{array}$$

Note that the derived problem (23) is non-convex, making it challenging to find the optimal solution. We next convert this problem into a linear programming problem by introducing a series of variables, which are defined as

(24) $$y_{q,m,l}^{s}=\pi_{F}\left(q,m\right)\eta_{l}f_{q,m,l}^{s}.$$

For the new variable, there is an intuitive understanding: the original variable $f_{q,m,l}^{s}$ is the probability of taking action s[t]=s when the queue state q[t]=q, time index m[t]=m, and channel state $h\left[t\right]=h_{l}$, whereas the new variable $y_{q,m,l}^{s}$ represents the probability of being in the queue state q[t]=q, time index m[t]=m, channel state $h\left[t\right]=h_{l}$, and taking action s[t]=s. Subsequently, we propose Theorem 2.

**Theorem** **2.** *The optimization problem (23) is equivalent to the following linear programming problem,*

(25a) $$\begin{array}{cc} \min_{\left\{y_{q,m,l}^{s}\right\}} & \sum_{q\in Q}\sum_{m\in M}\sum_{h_{l}\in H}\sum_{s\in S}P\left(s,l\right)y_{q,m,l}^{s} \end{array}$$

(25b) $$\begin{array}{cc} s.t. & \sum_{q\in Q}\sum_{m\in M}\sum_{h_{l}\in H}\sum_{s\in S}y_{q,m,l}^{s}1\left\{qT+mB>TB\right\}=0, \end{array}$$

(25c) $$\begin{array}{cc} \; & \sum_{q\in Q}\sum_{m\in M}\sum_{h_{l}\in H}\sum_{s\in S}y_{q,m,l}^{s}=1, \end{array}$$

(25d) $$\begin{array}{cc} \; & \sum_{q\in Q}\sum_{m\in M}\sum_{h_{l}\in H}\sum_{s\in S}{\tilde{\lambda }}_{\left(q,m\right),\left(\tilde{q},\tilde{m}\right)}y_{q,m,l}^{s}=\sum_{h_{l}\in H}\sum_{s\in S}y_{\tilde{q},\tilde{m},l}^{s},\forall \tilde{q}\in Q,\tilde{m}\in M,h_{l}\in H \end{array}$$

(25e) $$\begin{array}{cc} \; & y_{q,m,l}^{s}\geq 0, \end{array}$$

*where we define*

(26) $${\tilde{\lambda }}_{\left(q,m\right),\left(\tilde{q},\tilde{m}\right)}=\left\{\begin{array}{cc} 1\left\{\tilde{q}=q-s\right\}1\left\{\tilde{m}=m+1\right\} & \mathrm{if}\;m<T-1,\\ 1\left\{\tilde{q}=B\right\}1\left\{\tilde{m}=0\right\} & \mathrm{if}\;m=T-1. \end{array}\right.$$

**Proof.**  The proof starts with the observation that the objective functions of optimization problems (23) and (25) can be directly transformed into each other. The constraints (23b)–(23d) and (25b)–(25c) can be mutually converted using Equation (24) with the help of each other. The conversion of the transition equations, however, is more complex. Next, we focus on proving that the transition equations remain linear after the variable combination through Equation (24) and can be mutually converted. Then, when m<T−1, we have

(27a) $$\begin{array}{cc} \lambda_{\left(q,m\right),\left(\tilde{q},\tilde{m}\right)} & =\sum_{h_{l}\in H}\eta_{l}f_{q,m,l}^{s}1\left\{\tilde{q}=q-s\right\}1\left\{\tilde{m}=m+1\right\} \end{array}$$

(27b) $$\begin{array}{cc} \; & =\sum_{h_{l}\in H}\sum_{s\in S}\eta_{l}f_{q,m,l}^{s}1\left\{\tilde{q}=q-s\right\}1\left\{\tilde{m}=m+1\right\} \end{array}$$

(27c) $$\begin{array}{cc} \; & =\sum_{h_{l}\in H}\sum_{s\in S}\eta_{l}f_{q,m,l}^{s}{\tilde{\lambda }}_{\left(q,m\right),\left(\tilde{q},\tilde{m}\right)}. \end{array}$$

When m=T−1, we have

(28a) $$\begin{array}{cc} \lambda_{\left(q,m\right),\left(\tilde{q},\tilde{m}\right)} & =1\left\{\tilde{q}=B\right\}1\left\{\tilde{m}=0\right\} \end{array}$$

(28b) $$\begin{array}{cc} \; & =\sum_{h_{l}\in H}\sum_{s\in S}\eta_{l}f_{q,m,l}^{s}1\left\{\tilde{q}=B\right\}1\left\{\tilde{m}=0\right\} \end{array}$$

(28c) $$\begin{array}{cc} \; & =\sum_{h_{l}\in H}\sum_{s\in S}\eta_{l}f_{q,m,l}^{s}{\tilde{\lambda }}_{\left(q,m\right),\left(\tilde{q},\tilde{m}\right)}. \end{array}$$

Next, Equations (27) and (28) yield

(29a) $$\begin{array}{cc} \sum_{q\in Q}\sum_{m\in M}\lambda_{\left(q,m\right),\left(\tilde{q},\tilde{m}\right)}\pi_{F}\left(q,m\right) & =\sum_{q\in Q}\sum_{m\in M}\sum_{h_{l}\in H}\sum_{s\in S}\eta_{l}f_{q,m,l}^{s}{\tilde{\lambda }}_{\left(q,m\right),\left(\tilde{q},\tilde{m}\right)}\pi_{F}\left(q,m\right) \end{array}$$

(29b) $$\begin{array}{cc} \; & =\sum_{q\in Q}\sum_{m\in M}\sum_{h_{l}\in H}\sum_{s\in S}{\tilde{\lambda }}_{\left(q,m\right),\left(\tilde{q},\tilde{m}\right)}y_{q,m,l}^{s}. \end{array}$$

Then,

(30a) $$\begin{array}{cc} \pi_{F}\left(\tilde{q},\tilde{m}\right) & =\pi_{F}\left(\tilde{q},\tilde{m}\right)\sum_{h_{l}\in H}\sum_{s\in S}\eta_{l}f_{\tilde{q},\tilde{m},l}^{s} \end{array}$$

(30b) $$\begin{array}{cc} \; & =\sum_{h_{l}\in H}\sum_{s\in S}\eta_{l}f_{\tilde{q},\tilde{m},l}^{s}\pi_{F}\left(\tilde{q},\tilde{m}\right) \end{array}$$

(30c) $$\begin{array}{cc} \; & =\sum_{h_{l}\in H}\sum_{s\in S}y_{\tilde{q},\tilde{m},l}^{s}. \end{array}$$

Thus, we have proven that the two optimization problems can be mutually converted.    □

The solutions $\pi_{F}\left(q,m\right)$ and $f_{q,m,l}^{s}$ of optimization problem (23) can generate the solutions $y_{q,m,l}^{s}$ of optimization problem (25) according to Equation (24). Similarly, $y_{q,m,l}^{s}$ in problem (25) generates $\pi_{F}\left(q,m\right)$ by

(31) $$\pi_{F}\left(q,m\right)=\sum_{h_{l}\in H}\sum_{s\in S}y_{q,m,l}^{s}.$$

Likewise, we have

(32) $$f_{q,m,l}^{s}=\left\{\begin{array}{cc} \frac{y_{q,m,l}^{s}}{\pi_{F}\left(q,m\right)} & \mathrm{if}\;\pi_{F}\left(q,m\right)>0,\\ 1\left\{s=0\right\} & \mathrm{otherwise}. \end{array}\right.$$

We have demonstrated that if $\left(\pi_{F}\left(q,m\right),f_{q,m,l}^{s}\right)$ constitutes an optimal solution to problem (23), then the corresponding $y_{q,m,l}^{s}$ is an optimal solution to problem (25), and vice versa.

The attentive reader should have noticed that we are optimizing power consumption given *B* and *T*. The user-perceived capacity, on the other hand, is the capacity of the wireless communication system under a given power consumption. Therefore, we propose Algorithm 1 to address this issue, that is, to find the user-perceived capacity under a given power consumption. Meanwhile, the complexity of Algorithm 1 is $O\left(\log\left(\frac{P_{ave}T}{\Delta \;P_{\min}}\right)\right)$, where $\Delta \;P_{\min}=\min_{s,h_{l}}P\left(s+1,h_{l}\right)-P\left(s,h_{l}\right)$.
**Algorithm 1** User-Perceived Capacity Search Algorithm  1:**Input**: *T*, $P_{ave}$.  2:$B_{1}\leftarrow 0$, $B_{2}\leftarrow \frac{P_{ave}T}{\Delta \;P_{\min}}$  3:$B\leftarrow \left\lfloor \frac{\left(B_{1}+B_{2}\right)}{2}\right\rfloor$  4:P(B)← the optimal objective function of problem (25) with the frame size as *B*  5:P(B+1)← the optimal objective function of problem (25) with the frame size as B+1  6:**while** $P\left(B\right)>P_{ave}$ or $P\left(B+1\right)<P_{ave}$ **do**  7:    **if** $P\left(B\right)>P_{ave}$ **then**  8:        $B_{2}\leftarrow B$  9:    **else**10:        $B_{1}\leftarrow B$11:    **end if**12:    $B\leftarrow \left\lfloor \frac{\left(B_{1}+B_{2}\right)}{2}\right\rfloor$13:    P(B)← the optimal objective function of problem (25) with the frame size as *B*14:    P(B+1)← the optimal objective function of problem (25) with the frame size as B+115:**end while**16:$\frac{B}{T}$ is the maximum user-perceived capacity.

## 5. The Structure of the Optimal Policy

In this section, we develop a series of theoretical results for the structural properties of the optimal scheduling policy. Note that *B* packets arriving at the beginning of each period must be transmitted within one arrival period. Thus, we only need to consider the data transmission for one period, i.e., *T* time-slots.

Within one period, we first represent the constraint on the user-perceived capacity, i.e., Equation (25b) by introducing the upper and lower bounds of the queue length. In particular, they are expressed as

(33) $$\left\{\begin{array}{cc} q\left[m\right]\leq \;\left\lfloor B-\frac{Bm}{T}\right\rfloor & \mathrm{upper}\;\mathrm{bound},\\ q\left[m\right]\geq \;{\left(B-Sm\right)}^{+} & \mathrm{lower}\;\mathrm{bound}, \end{array}\right.$$

through which we define $q_{m+1}^{\mathrm{lb}}={\left(B-S(m+1)\right)}^{+}$ and $q_{m+1}^{\mathrm{ub}}=\left\lfloor B-\frac{B(m+1)}{T}\right\rfloor$. Consequently, the feasible region of the transmission rate in time-slot *m* is given by

(34) $$S_{m}\left(q\left[m\right]\right)=\left\{s\left[m\right]\in \left\{0,1,\ldots ,S\right\}\;|\;\max\left\{q\left[m\right]-q_{m+1}^{\mathrm{ub}},0\right\}\leq \;s\left[m\right]\leq \;\min\left\{q\left[m\right],S\right\}\right\}.$$

Furthermore, we represent the data transmission as a finite horizon Markov decision process, which is constructed as follows:

- **State:** The pair of the queue length and channel state (q[m],h[m]), where $q_{m}^{\mathrm{lb}}\leq \;q\left[m\right]\leq \;q_{m}^{\mathrm{ub}}$ and $h\left[m\right]\in \left\{h_{1},\ldots ,h_{L}\right\}$ with $h_{1}<h_{2}<\cdots <h_{L}$.
- **Action:** The transmission rate $s\left[m\right]\in S_{m}\left(q\left[m\right]\right)$.
- **Transition:** The queue length evolves according to Equation (6) and the channel state is *i.i.d.* over time, following the probability distribution $\left\{\eta_{1},\ldots ,\eta_{L}\right\}$.
- **Value function:**
  (35) $$V_{m}^{*}\left(q,l\right)=\min_{s\in S_{m}\left(q\right)}P\left(s,l\right)+\sum_{l=1}^{L}\eta_{l}\;V_{m+1}^{*}\left(q-s,l\right),\;\;\;m=0,1,\ldots ,T-1,$$
  where $V_{T}^{*}\left(q,l\right)=0$ for each *q* and *l*.
- **Scheduling policy:**
  (36) $$s_{m}^{*}\left(q,l\right)=\arg\min_{s\in S_{m}\left(q\right)}Q_{m}^{*}\left(q,l,s\right)=\arg\min_{s\in S_{m}\left(q\right)}P\left(s,l\right)+\sum_{l=1}^{L}\eta_{l}\;V_{m+1}^{*}\left(q-s,l\right),$$
  where the item $P\left(s,l\right)+\sum_{l=1}^{L}\eta_{l}\;V_{t}^{*}\left(q-s,l\right)$ is referred to as the Q-value function, denoted by $Q_{m}^{*}\left(q,l,s\right)$. According to theoretical results for finite horizon MDPs [66], considering the scheduling policy in (36) will not lose the optimality for the average minimizing problem (12). Furthermore, the policy in (36) can be re-written in the form of the probabilistic policy F in Section 3.1 by setting $f_{q,m,l}^{s}=1\left\{s=s_{m}^{*}\left(q,l\right)\right\}$.

Then, we solve the finite horizon MDP by the dynamic programming in Algorithm 2 with the complexity $O({\left(B+1\right)}^{2}L^{2}\left(S+1\right)T)$. By analyzing Algorithm 2, we further have the following theorem.
**Algorithm 2** Dynamic programming for finite horizon Markov decision processes  1:m←T−1  2:**for** $q_{m}^{\mathrm{lb}}\leq \;q\left[m\right]\leq \;q_{m}^{\mathrm{ub}}$, $h\left[m\right]\in \left\{h_{1},\ldots ,h_{L}\right\}$ **do**  3:    $V_{m}^{*}\left(q,l\right)=\min_{s\in S_{m}\left(q\right)}P\left(s,l\right)$  4:**end for**  5:**Recursion**: For each time step *m* from T−2 to 0, the value function $V_{m}^{*}\left(q,l\right)$ is updated using the Bellman equation.  6:**for** m∈{T−2,T−3,…,0} **do**  7:    $V_{m}^{*}\left(q,l\right)=\min_{s\in S_{m}\left(q\right)}P\left(s,l\right)+\sum_{l=1}^{L}\eta_{l}\;V_{m+1}^{*}\left(q-s,l\right)$  8:**end for**  9:**Policy Extraction**: After computing the value functions for all time steps, the optimal policy can be extracted by choosing the action that maximizes the value function at each state and time step.10:**for** m∈{T−1,T−2,…,0} **do**11:    **for** $q_{m}^{\mathrm{lb}}\leq \;q\left[m\right]\leq \;q_{m}^{\mathrm{ub}}$, $h\left[m\right]\in \left\{h_{1},\ldots ,h_{L}\right\}$ **do**12:        **for** $s\in S_{m}\left(q\right)$ **do**13:           $Q_{m}^{*}\left(q,l,s\right)\leftarrow P\left(s,l\right)+\sum_{l=1}^{L}\eta_{l}\;V_{m+1}^{*}\left(q-s,l\right)$14:        **end for**15:        $s_{m}^{*}\left(q,l\right)\leftarrow \arg\min_{s\in S_{m}\left(q\right)}Q_{m}^{*}\left(q,l,s\right)$16:    **end for**17:**end for**

**Theorem** **3.** The optimal scheduling $s_{m}^{*},\;m=0,\ldots ,T-1$, can be re-written as a series of thresholds $q_{m}^{\mathrm{th}}\left(l,s\right),\;\forall \;m,h,s$. That is,

(37) $$s_{m}^{*}\left(q,l\right)=s\;\;\;\mathrm{if}\;q_{m}^{\mathrm{th}}\left(l,s-1\right)<\;q\leq \;q_{m}^{\mathrm{th}}\left(l,s\right),$$

*where the thresholds satisfy $q_{m}^{\mathrm{th}}\left(l,s-1\right)\leq \;q_{m}^{\mathrm{th}}\left(l,s\right)$ for all s=0,…,S. Meanwhile, $q_{m}^{\mathrm{th}}\left(l,-1\right)\;=\;-1$ and $q_{m}^{\mathrm{th}}\left(l,S\right)=B$.*

**Proof.**  We prove the threshold-based structure of the optimal policy by induction. Assuming that $V_{m+1}^{*}\left(q,l\right)$ is convex over *q*, we demonstrate the thresholds of the optimal policy at time-slot *m*. After that, the threshold-based structure can induce to the convexity of $V_{m}^{*}\left(q,l\right)$. Specifically, the convexity of $V_{m+1}^{*}\left(q,l\right)$ holds for m=T−1 due to $V_{T}^{*}\left(q,l\right)=0$. For time-slot *m*, we first represent the threshold-based structure by the following two assertions:

(38) $$\begin{array}{cc} \; & Q_{m}^{*}\left(q,l,s^{*}\right)\leq \;Q_{m}^{*}\left(q,l,s^{*}-\delta \right)\Rightarrow Q_{m}^{*}\left(q+1,l,s^{*}\right)\leq \;Q_{m}^{*}\left(q+1,h,s^{*}-\delta \right), \end{array}$$

(39) $$\begin{array}{cc} \; & Q_{m}^{*}\left(q,l,s^{*}\right)\leq \;Q_{m}^{*}\left(q,l,s^{*}+\delta \right)\Rightarrow Q_{m}^{*}\left(q+1,l,s^{*}+1\right)\leq \;Q_{m}^{*}\left(q+1,h,s^{*}+1+\delta \right), \end{array}$$

where $s^{*}=s_{m}^{*}\left(q,l\right)=\arg\min_{s\in S_{m}\left(q\right)}Q_{m}^{*}\left(q,l,s\right)$. (For completeness, we set $Q_{m}^{*}\left(q,l,s\right)=+\infty$ for $s\notin \;S_{m}\left(q\right)$ and V(q,l)=+∞ for $q<q_{m}^{\mathrm{lb}}$ or $q>q_{m}^{\mathrm{ub}}$.) Based on the definition of the optimal policy in Equations (36), (38) and (39) implies that the optimal transmission rate $\tilde{s}=\arg\min_{s\in S_{m}\left(q+1\right)}Q_{m}^{*}\left(q+1,l,s\right)$ for the state (q+1,l) satisfies $s^{*}\leq \tilde{s}\leq \;s^{*}+1$. That is, the optimal transmission rate is monotonically increasing over *q* for each given *h* and *m*, through which we can derive the threshold-based structure in Theorem 1.

Next, we prove Equations (38) and (39) based on the convexity of $V_{t+1}^{*}\left(q,l\right)$ and P(s,l). For Equation (38), it suffices to show that

(40) $$Q_{m}^{*}\left(q+1,l,s^{*}\right)-Q_{m}^{*}\left(q,l,s^{*}\right)\leq \;Q_{m}^{*}\left(q+1,l,s^{*}-\delta \right)-Q_{m}^{*}\left(q,l,s^{*}-\delta \right)$$

when $Q_{m}^{*}\left(q,l,s^{*}\right)\leq \;Q_{m}^{*}\left(q,l,s^{*}-\delta \right)$. Note that $Q^{*}\left(q,l,s\right)=P\left(s,l\right)+\sum_{l=1}^{L}\eta_{l}\;V_{m+1}^{*}\left(q-s,l\right)$ is given based on the expression of $Q^{*}\left(q,l,s\right)$ in (36). As such, we can re-write (38) as

(41) $$\begin{array}{cc} \; & \sum_{l=1}^{L}\eta_{l}\;\left[\left(V_{m+1}^{*}\left(q-s^{*}+1,l\right)-V_{m+1}^{*}\left(q-s^{*},l\right)\right)\right]\\ \leq & \sum_{l=1}^{L}\eta_{l}\;\left[\left(V_{m+1}^{*}\left(q-s^{*}+\delta +1,l\right)-V_{m+1}^{*}\left(q-s^{*}+\delta ,l\right)\right)\right]. \end{array}$$

According to the convexity of $V^{*}\left(q,l\right)$, $V_{m+1}^{*}\left(q-s^{*}+1,l\right)-V_{m+1}^{*}\left(q-s^{*},l\right)\leq \;V_{m+1}^{*}\left(q-s^{*}+\delta +1,l\right)-V_{m+1}^{*}\left(q-s^{*}+\delta ,l\right)$ holds for any *l*. Based on this, Equation (38) has been proven.

Similarly, we demonstrate Equation (39) by considering its sufficient condition:

(42) $$Q_{m}^{*}\left(q+1,l,s^{*}+1\right)-Q_{m}^{*}\left(q,l,s^{*}\right)\leq \;Q_{m}^{*}\left(q+1,l,s^{*}+1+\delta \right)-Q_{m}^{*}\left(q,l,s^{*}+\delta \right).$$

when $Q_{m}^{*}\left(q,l,s^{*}\right)\leq \;Q_{m}^{*}\left(q,l,s^{*}+\delta \right)$. Note that $Q^{*}\left(q,l,s\right)=P\left(s,l\right)+\sum_{l=1}^{L}\eta_{l}\;V_{m+1}^{*}\left(q-s,l\right)$ is given based on the expression of $Q^{*}\left(q,l,s\right)$ in (36). As such, we can re-write (39) as

(43) $$\begin{array}{cc} \; & P\left(s^{*}+1,l\right)-P\left(s^{*},l\right)+\sum_{l=1}^{L}\eta_{l}\;\left[\left(V_{m+1}^{*}\left(q-s^{*},l\right)-V_{m+1}^{*}\left(q-s^{*},l\right)\right)\right]\\ \leq & P\left(s^{*}+1+\delta ,l\right)-P\left(s^{*}+\delta ,l\right)+\sum_{l=1}^{L}\eta_{l}\;\left[\left(V_{m+1}^{*}\left(q-s^{*}-\delta ,l\right)-V_{m+1}^{*}\left(q-s^{*}-\delta ,l\right)\right)\right]. \end{array}$$

Consequently, it is equivalent to

(44) $$P\left(s^{*}+1,l\right)-P\left(s^{*},l\right)\leq \;P\left(s^{*}+1+\delta ,l\right)-P\left(s^{*}+\delta ,l\right).$$

This assertion is true due to the convexity of P(s,l) over *s*.

Finally, we prove the convexity of $V_{m}^{*}\left(q,l\right)$ over *q*, i.e.,

(45) $$2V_{m}^{*}\left(q,l\right)\leq \;V_{m}^{*}\left(q+1,l\right)+V_{m}^{*}\left(q-1,l\right).\;$$

Recalling that $s^{*}=s_{m}^{*}\left(q,l\right)$, we have $V_{m}^{*}\left(q,l\right)=Q\left(q,l,s^{*}\right)$. Likewise, we represent $\tilde{s}=s_{m}^{*}\left(q+1,l\right)$ and $\overset{˘}{s}=s_{m}^{*}\left(q-1,l\right)$. According to the threshold-based structure, illustrated by Equations (38) and (39), we have

(46) $$s^{*}\leq \;\tilde{s}\leq \;s^{*}+1\;\;\;\;\mathrm{and}\;\;\;\;s^{*}-1\leq \overset{˘}{s}\leq \;s^{*}.\;$$

This way, four potential cases for the values of $s^{*}$, $\tilde{s}$, and $\overset{˘}{s}$ are listed as follows: (1) $\tilde{s}=s^{*}$ and $\overset{˘}{s}=s^{*}$; (2) $\tilde{s}=s^{*}+1$ and $\overset{˘}{s}=s^{*}$; (3) $\tilde{s}=s^{*}$ and $\overset{˘}{s}=s^{*}-1$; and (4) $\tilde{s}=s^{*}+1$ and $\overset{˘}{s}=s^{*}-1$. For the first three cases, we prove Equation (45) by establishing an upper bound of its left-hand side, i.e.,

(47) $$\begin{array}{cc} 2V_{m}^{*}\left(q,l\right)\leq \; & Q_{m}^{*}\left(q,l,\tilde{s}\right)+Q_{m}^{*}\left(q,l,\overset{˘}{s}\right)\\ = & P\left(\tilde{s},l\right)+P\left(\overset{˘}{s},l\right)+\sum_{l=1}^{L}\eta_{l}\left(V_{t+1}^{*}\left(q-\tilde{s},l\right)+V_{m+1}^{*}\left(q-\overset{˘}{s},l\right)\right) \end{array}$$

Meanwhile, we have

(48) $$\begin{array}{cc} \; & V_{m}^{*}\left(q+1,l\right)+V_{m}^{*}\left(q-1,l\right) \end{array}$$

(49) $$\begin{array}{cc} \; & =P\left(\tilde{s},l\right)+P\left(\overset{˘}{s},l\right)+\sum_{l=1}^{L}\eta_{l}\left(V_{m+1}^{*}\left(q+1-\tilde{s},l\right)+V_{m+1}^{*}\left(q-1-\overset{˘}{s},l\right)\right). \end{array}$$

Based on the convexity of $V_{m+1}^{*}\left(q,l\right)$, we have

(50) $$V_{m+1}^{*}\left(q-\tilde{s},l\right)+V_{m+1}^{*}\left(q-\overset{˘}{s},l\right)\leq \;V_{m+1}^{*}\left(q+1-\tilde{s},l\right)+V_{m+1}^{*}\left(q-1-\overset{˘}{s},l\right)\;$$

when $\overset{˘}{s}\geq \;\tilde{s}-1$. For the last case, i.e., $\tilde{s}=s^{*}+1$ and $\overset{˘}{s}=s^{*}-1$, we prove Equation (45) by

(51) $$\begin{array}{cc} \; & 2V_{m}^{*}\left(q,l\right)-V_{m}^{*}\left(q+1,l\right)-V_{m}^{*}\left(q-1,l\right)\\ = & 2P\left(s^{*},l\right)-P\left(s^{*}+1,l\right)-P\left(s^{*}-1,l\right)\\ \; & +\sum_{l=1}^{L}\eta_{l}\left(2V_{m+1}^{*}\left(q-s^{*},l\right)-V_{m+1}^{*}\left(q+1-\left(s^{*}+1\right),l\right)-V_{m+1}^{*}\left(q-1-\left(s^{*}-1\right),l\right)\right)\\ = & 2P\left(s^{*},l\right)-P\left(s^{*}+1,l\right)-P\left(s^{*}-1,l\right)\geq \;0 \end{array}$$

The equality is given by substituting the definition of $V_{m}^{*}\left(q,l\right)$ in Equation (45). Meanwhile, the inequality holds due to the convexity of P(s,l) over *s*.

Overall, we first assumed the convexity of the value function and proved that the optimal policy has a threshold structure. Then, we established the convexity of the value function through mathematical induction. Consequently, we have completed the proof that the optimal policy from the perspective of the queue state possesses a threshold structure.    □

Then, we reveal the threshold structure from over the time index m[t] as follows.

**Lemma** **2.** $V_{m}^{*}\left(q,l\right)$ monotonically increases over q.

**Proof.**  We assume $V_{m+1}^{*}\left(q+1,l\right)\geq \;V_{m+1}^{*}\left(q,l\right)$ inductively. It is true for m=T−1 since $V_{T}^{*}\left(q,l\right)=0$. Next, we have

(52) $$\begin{array}{cc} V_{m}^{*}\left(q+1,l\right)-V_{m}^{*}\left(q,l\right)\geq \; & Q_{m}^{*}\left(q+1,l,\tilde{s}\right)-Q_{m}^{*}\left(q,l,\tilde{s}\right)\\ = & \sum_{l=1}^{L}\eta_{l}\left(V_{m+1}^{*}\left(q-\tilde{s}+1,l\right)-V_{m+1}^{*}\left(q-\tilde{s},l\right)\right)\geq \;0, \end{array}$$

where $\tilde{s}=s_{m}^{*}\left(q+1,l\right)$. As such, the first equality holds since $V_{m}^{*}\left(q+1,l\right)=Q_{m}^{*}\left(q+1,l,\tilde{s}\right)$ and $V_{m}^{*}\left(q,l\right)\leq \;Q_{m}^{*}\left(q,l,\tilde{s}\right)$. This way, we prove this lemma.    □

**Theorem** **4.** *The thresholds of the optimal scheduling policy under different times satisfy*

(53) $$q_{m+1}^{\mathrm{th}}\left(l,s\right)\leq \;q_{m}^{\mathrm{th}}\left(l,s\right),\;\;\;\forall m=0,\ldots ,T-2.\;$$

**Proof.**  We indicate that the optimal transmission rate increases with time-slot *m* in this theorem, i.e., $s_{m}^{*}\left(q,l\right)\leq \;s_{m+1}^{*}\left(q,l\right)$.

Furthermore, we give an intuitive explanation of how the optimal transmission rate is selected. In particular, we have

(54) $$s_{m}^{*}\left(q,l\right)=\arg\min_{s\in S_{m}\left(q\right)}P\left(s,l\right)+{\bar{V}}_{m+1}^{*}\left(q-s\right),$$

where

(55) $${\bar{V}}_{m}^{*}\left(q\right)=\sum_{l=1}^{L}\eta_{l}V_{m}^{*}\left(q,l\right).$$

Given the values of *q* and *l*, we note that P(s,l) is increasing and convex over *s*, while ${\bar{V}}_{m+1}^{*}\left(q-s\right)$ is decreasing and convex over *s* based on Theorem 1 and Lemma 2. This way, we can represent the optimal transmission rate as

(56) $$s_{m}^{*}\left(q,l\right)=\max\left\{s\in S_{m}\left(q\right)\;|\;P\left(s,l\right)-P\left(s-1,l\right)\leq \;{\bar{V}}_{m+1}^{*}\left(q-s+1\right)-{\bar{V}}_{m+1}^{*}\left(q-s\right)\right\}.$$

As a result, we have $s_{m+1}^{*}\left(q,l\right)\geq \;s_{m}^{*}\left(q,l\right)$ when

(57) $${\bar{V}}_{m}^{*}\left(q+1\right)-{\bar{V}}_{m}^{*}\left(q\right)\leq \;{\bar{V}}_{m+1}^{*}\left(q+1\right)-{\bar{V}}_{m+1}^{*}\left(q\right),\;\;\;\;\forall \;m=0,\ldots ,T-2.$$

This statement holds based on two observations: (1) $S_{m}\left(q\right)$ removes the lower transmission rates when *m* is increasing, which is due to the monotonicity of $q_{m}^{\mathrm{ub}}$; (2) for each *s* satisfying $P\left(s,l\right)-P\left(s-1,l\right)\leq \;{\bar{V}}_{m+1}^{*}\left(q-s+1\right)-{\bar{V}}_{m+1}^{*}\left(q-s\right)$, we have $P\left(s,l\right)-P\left(s-1,l\right)\leq {\bar{V}}_{m+2}^{*}\left(q-s+1\right)-{\bar{V}}_{m+2}^{*}\left(q-s\right)$ as per (57).

Since ${\bar{V}}_{m}^{*}\left(q\right)=\sum_{l=1}^{L}\eta_{l}V_{m}^{*}\left(q,l\right)$, we show (57) by the following sufficient condition:

(58) $$\begin{array}{cc} V_{m}^{*}\left(q+1,l\right)-V_{m}^{*}\left(q,l\right) & \leq \;P\left(s^{*},l\right)-P\left(s^{*},l\right)+\sum_{l=1}^{L}\eta_{l}\left(V_{m+1}^{*}\left(q+1-s^{*},l\right)-V_{m+1}^{*}\left(q-s^{*},l\right)\right)\\ \; & \leq \;\sum_{l=1}^{L}\eta_{l}\left(V_{m+1}^{*}\left(q+1,l\right)-V_{m+1}^{*}\left(q,l\right)\right)\\ \; & ={\bar{V}}_{m+1}^{*}\left(q+1\right)-{\bar{V}}_{m+1}^{*}\left(q\right). \end{array}$$

where we recall that $s^{*}=s_{m}^{*}\left(q,l\right)$. Meanwhile, the last inequality holds due to the convexity of $V_{m+1}^{*}\left(q,l\right)$ over *q*.    □

Then, we reveal the threshold structure from over the channel state h[t] as follows.

**Theorem** **5.** *If the power consumption function P(s,h) satisfies*

(59) $$P\left(s+1,l^{+}\right)-P\left(s,l^{+}\right)\leq \;P\left(s+1,l^{-}\right)-P\left(s,l^{-}\right),\;\;\;\forall \;s=0,\ldots ,S-1,$$

*for the case when $l^{+}>l^{-}$, the order relation of thresholds over different channel states is*

(60) $$q_{m}^{\mathrm{th}}\left(l^{+},s\right)\leq \;q_{m}^{\mathrm{th}}\left(l^{-},s\right).$$

**Proof.**  We start the proof by noticing that $q_{m}^{\mathrm{th}}\left(l^{+},s\right)\leq \;q_{m}^{\mathrm{th}}\left(l^{-},s\right)$ means that a larger transmission rates is obtained for queue length *q* when the system suffers a higher channel gain. That is, $s_{m}^{*}\left(q,l^{+}\right)\geq \;s_{m}^{*}\left(q,l^{-}\right)$. To prove this, we represent $\tilde{s}=s_{m}^{*}\left(q,l^{+}\right)$ and $\overset{˘}{s}=s_{m}^{*}\left(q,l^{-}\right)$ and give an equivalent expression of $\tilde{s}\geq \;\overset{˘}{s}$ as

(61) $$Q_{m}^{*}\left(q,l^{-},\overset{˘}{s}\right)\leq \;Q_{m}^{*}\left(q,l^{-},\overset{˘}{s}-\delta \right)\Rightarrow Q_{m}^{*}\left(q,l^{+},\overset{˘}{s}\right)\leq \;Q_{m}^{*}\left(q,l^{+},\overset{˘}{s}-\delta \right).$$

This implies that the optimal transmission rate for the state $\left(q,l^{+}\right)$ shall be selected from the set $S_{m}\left(q\right)\cap \left\{\overset{˘}{s},\overset{˘}{s}+1,\ldots ,S\right\}$, i.e., $\tilde{s}\geq \;\overset{˘}{s}$. We next show (61) by considering its sufficient condition:

(62) $$\begin{array}{cc} \; & Q_{m}^{*}\left(q,l^{+},\overset{˘}{s}\right)+Q_{m}^{*}\left(q,l^{-},\overset{˘}{s}-\delta \right)-Q_{m}^{*}\left(q,l^{+},\overset{˘}{s}-\delta \right)-Q_{m}^{*}\left(q,l^{-},\overset{˘}{s}\right)\\ = & P\left(\overset{˘}{s},l^{+}\right)+P\left(\overset{˘}{s}-\delta ,l^{-}\right)-P\left(\overset{˘}{s}-\delta ,l^{+}\right)-P\left(\overset{˘}{s},l^{-}\right)\leq \;0, \end{array}$$

where the inequality holds due to (59).    □

By exploiting Theorems 3–5, we finally develop an efficient method in Algorithm 3 to search thresholds for the optimal transmission policy. As presented in Algorithm 3, we can reduce the running time to update the optimal transmission rate based on the monotonicity of $s_{m}^{*}\left(q,l\right)$ over *q* in line 5. Similarly, given $q_{m}^{\mathrm{th}}\left(l,s\right)$ for specific *l* and *m*, we can constrain the transmission rates of states with $\tilde{l}<l$ and $\tilde{m}<m$ in lines 9 and 11. As such, the complexity of Algorithm 3 is O(S+1) in the best case, while it is equal to $O({\left(B+1\right)}^{2}L^{2}\left(S+1\right)T)$ in the worst case, the same as that in Algorithm 2.
**Algorithm 3** Algorithm to search thresholds for the optimal transmission policy  1:$q_{T-1}^{\mathrm{th}}\left(1,s\right)\leftarrow \;\left(B+1\right)1_{\left\{s=S\right\}}-1$ for all *s*  2:**for** m=T−1 to 0 **do**  3:    **for** l=L to 1 **do**  4:        **for** $q=q_{m}^{\mathrm{ub}}$ to $q_{m}^{\mathrm{lb}}$ **do**  5:           $s_{m}^{*}\left(q,l\right)\leftarrow \arg\min_{s\in S_{m}\left(q\right)\cap \left\{s\leq \min\left\{\overset{˘}{s}|q\leq \;q_{m}^{\mathrm{th}}\left(l,\overset{˘}{s}\right)\right\}\right\}}Q_{m}^{*}\left(q,l,s\right)$  6:           $q_{m}^{\mathrm{th}}\left(l,s_{m}^{*}\left(q,l\right)\right)\leftarrow \;q$  7:           $V_{m}^{*}\left(q,l\right)\leftarrow \;Q_{m}^{*}\left(q,l,s_{m}^{*}\left(q,l\right)\right)$  8:        **end for**  9:        $q_{m}^{\mathrm{th}}\left(\tilde{l},s\right)\leftarrow \;q_{m}^{\mathrm{th}}\left(l,s\right)$ for all $\tilde{l}<l$10:    **end for**11:    $q_{\tilde{m}}^{\mathrm{th}}\left(l,s\right)\leftarrow \;q_{m}^{\mathrm{th}}\left(l,s\right)$ for all $\tilde{m}<m$12:**end for**

## 6. Simulation Results

In this section, we will validate the accuracy and effectiveness of our theoretical results through simulation. We assume that the large scale path loss is calculated as $\rho =28+22\log_{10}d+20\log_{10}f_{c}\;$dB with the carrier frequency $f_{c}$ as 6GHz and the distance *d* as 100m, grounded in a typical channel model in the 5G NR standard [67]. Next, we calculate the transmission powers over the AWGN channel as P(0)=0 W, P(1)=5.19mW, and P(2)=10.38mW by using BPSK and QPSK modulations when transmission rate s[t]=1 and 2, respectively. In particular, the occupied bandwidth is 1 MHz and the one-sided noise power spectral density $N_{0}$ is −150 dBm/Hz. Additionally, we model the fading channel with a three-state block-fading distribution, with states [1,4,9] and equal probabilities. The power consumption over fading channels is equal to $P\left(s,l\right)=\frac{P(s)}{h_{l}^{2}}$.

**Figure 3:**
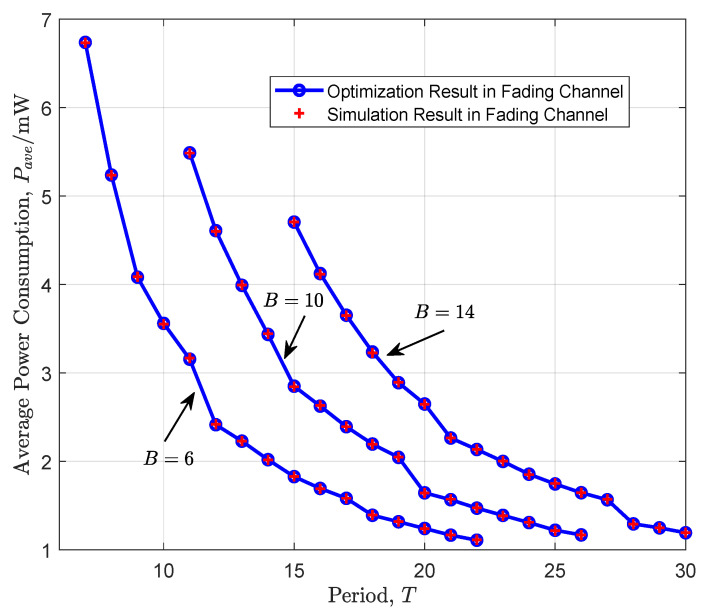
The power–period tradeoff in a fading channel.

Figure 3 demonstrates the performance of the policy in the fading channel scenario. Since we adopt discrete time-slots, only the points shown in the figure represent the operating points of our proposed policy. The horizontal axis represents the period size, which is the maximum number of service time-slots for the periodically arriving data packets. The vertical axis represents the average power consumption. We demonstrate the performance of cross-layer scheduling with B=6, 10, and 14, where the frame sizes are quantized by the minimum transmission unit $\delta =5\times 10^{3}\;$ bits. Meanwhile, we set T∈{7,…,30}, with the timeslot duration as 0.5ms. We observe that the theoretical results of the proposed policy align well with the simulation results, providing a cross-layer scheduling policy that meets the continuous reading requirement in the fading channel scenario. We also notice that, when the periodically arriving data packets are fixed, the average power consumption decreases as period *T* increases. There are two reasons for this. First, similar to the AWGN scenario, as the period increases, the average power consumption naturally decreases. Second, a larger period means more time-slots are available for transmission, allowing for the possibility to wait for better channel conditions, thereby reducing power consumption.

**Figure 4:**
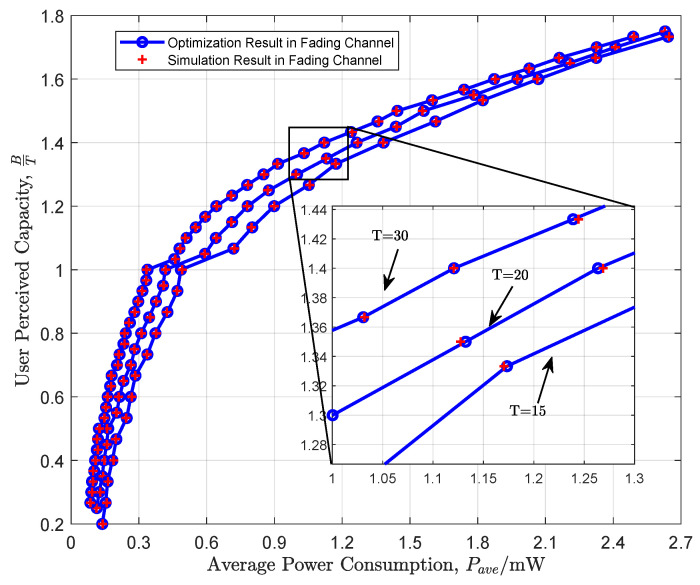
User-perceived capacity in fading channel.

Figure 4 illustrates the variation of user-perceived capacity with respect to the average power consumption in a fading channel scenario. The horizontal axis represents the average power consumption, while the vertical axis denotes the user-perceived capacity. We present performance variations for the cases of T=15,20,30. It is observed that there is good agreement between theoretical and simulated results. Additionally, it is noted that as the average power increases, the user-perceived capacity continuously increases. However, the rate of increase in user-perceived capacity slows down with the increase in average power. This is consistent with the law of channel capacity, where the marginal effect of the capacity increase diminishes as the average power increases.

**Figure 5:**
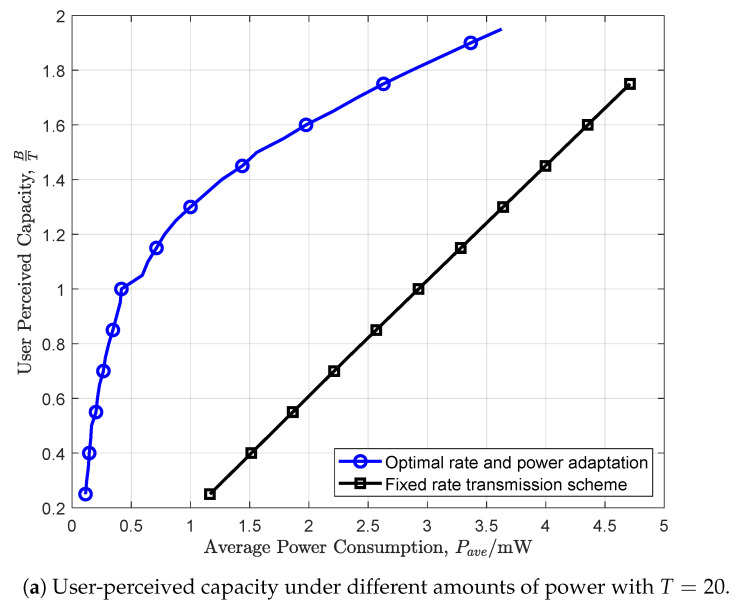

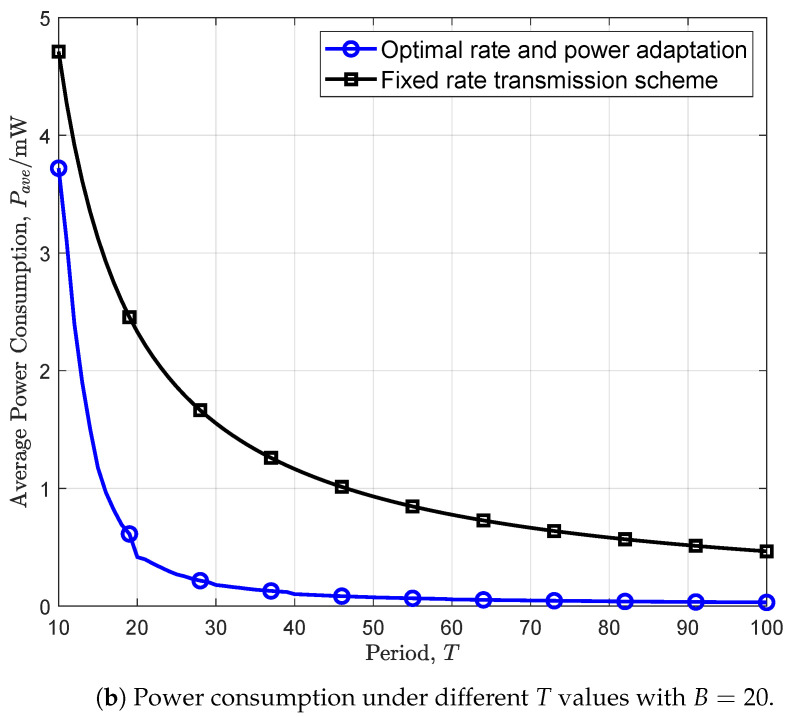
Comparisons of the optimal scheme with a fixed rate transmission scheme.

We next compare the optimal rate and power adaptation scheme with a heuristic method, referred to as the fixed rate transmission scheme. Specifically, this benchmark serves the user with a fixed rate B/T in each timeslot. In Figure 5a, we set T=20 and evolve the user-perceived capacity under the two schemes. It is evident that the user-perceived capacity achievable by our policy is higher than the fixed rate transmission scheme. Similarly, Figure 5b presents the average power under different values of *T* with B=20. Since the benchmark dose not consider the time-varying channel state, it suffers from a larger amount of power consumption compared to the optimal scheme.

**Figure 6:**
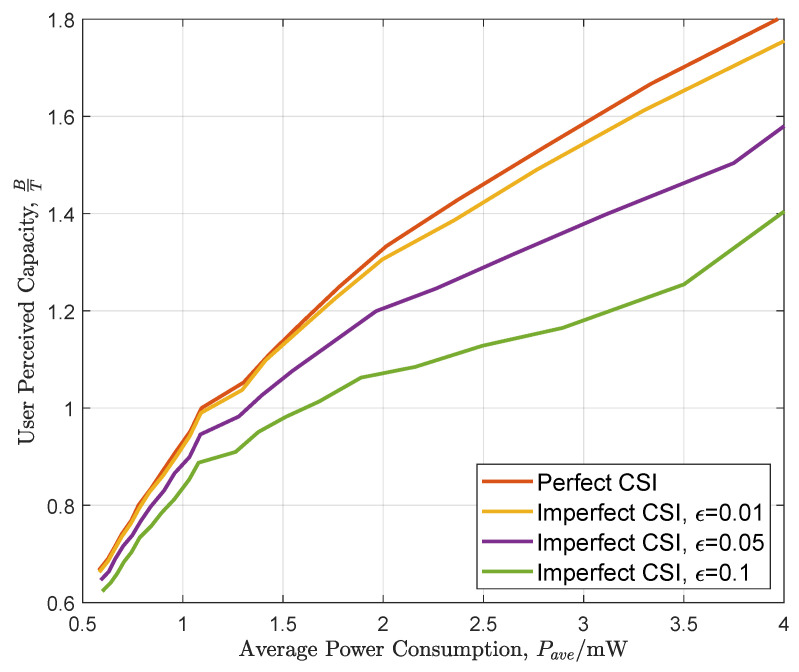
The influence of imperfect CSI for user-perceived capacity.

Figure 6 demonstrates the influence of the imperfect channel state information to the performance of the proposed transmission scheme, where we set B=20 and T∈{10,20}. Specifically, ϵ indicates the probability that the actual channel state is not equal to the estimated channel state. In this case, the estimated channel state is randomly selected among all the possible values with equal probabilities. As shown in this figure, the user-perceived capacity will decrease with the increment of ϵ.

Additionally, Table 1 displays the average running times of Algorithm 2 and the Algorithm 3 under different values of *B* and *T*. Notably, Algorithm 3 exhibits a significant reduction in running time compared to the dynamic programming in Algorithm 2.

**Figure 7:**
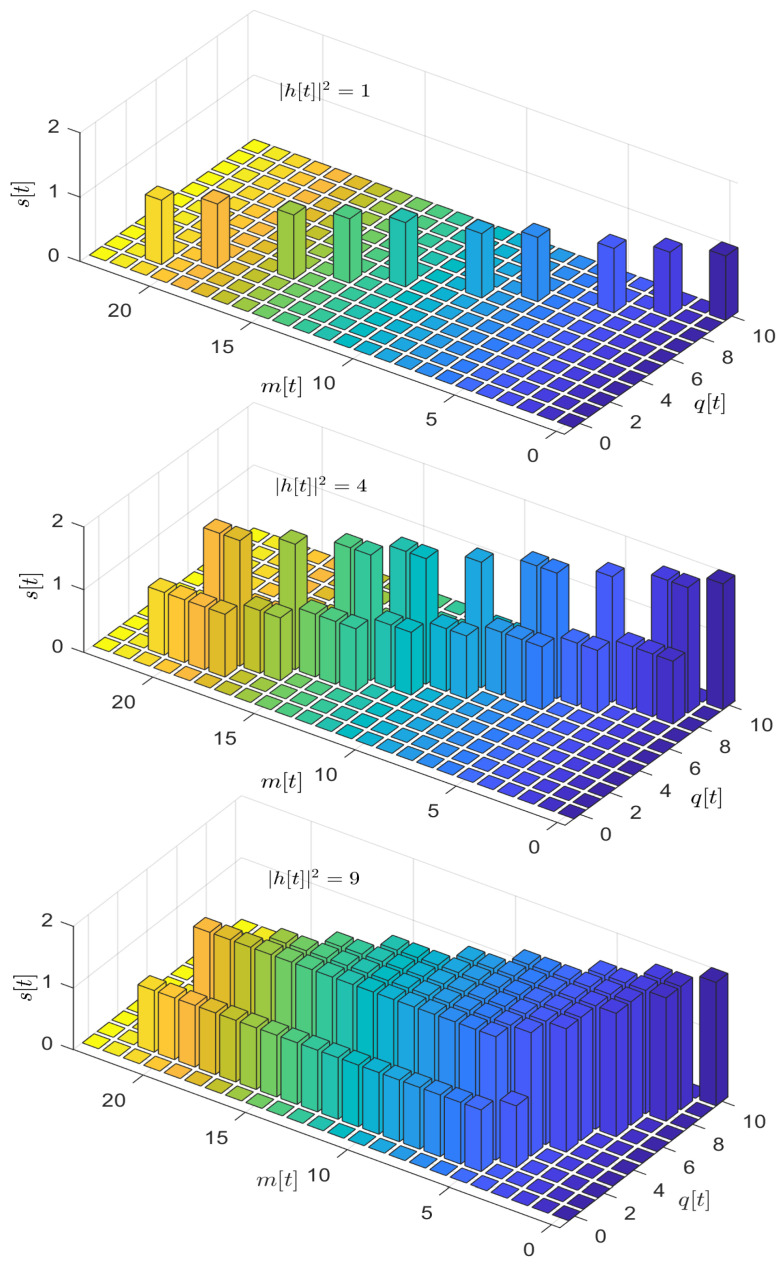
The threshold structure of the optimal policy: the three subfigures present the optimal policy with ${\left|h\left[t\right]\right|}^{2}=1,4,\;\mathrm{and}\;9$, successively.

Figure 7 illustrates the threshold structure of the optimal policy. In this simulation, we set the number of periodically arriving data packets to B=10 and the number of time-slots in a period to T=24. Subplots 1 through 3 represent the optimal scheduling policy under three different channel states, respectively. We observe that within the feasible space (q,m)∈{(q,m)|qT+mB≤TB,B−q≤mS}, the optimal policy exhibits a threshold structure, validating the queue length-based optimal policy structure proposed earlier. Additionally, we note that as the channel condition improves, the transmission rate increases, confirming the channel-based optimal policy structure we proposed.

## 7. Conclusions

This paper has focused on the concept of user-perceived throughput, a crucial performance metric that encapsulates the essence of the user experience in mobile multimedia services. Our study has considered the impact of channel state information, both at the receiver (CSIR) and the transmitter (CSIT), on enhancing the user-perceived capacity. It is observed that the capacity is severely limited when only CSIR is available, potentially reaching a lower bound of zero. In contrast, the integration of CSIT with CSIR facilitates substantial improvements in the user-perceived capacity associated with adaptive power and rate strategies. We have proposed an MDP-based methodology for calculating the user-perceived capacity under a cross-layer design of power and rate adaption. Based on this, we have solved an average power minimization problem with a constraint on the user-perceived capacity by converting it as linear programming. Consequently, the maximum user-perceived capacity under different power consumption amounts has been calculated based on a binary searching method. Furthermore, our findings have indicated that the optimal cross-layer policy adheres to a threshold-based structure dependent on time, backlog, and channel gain. Additionally, when power adaptation is implemented as the sole policy, the user-perceived capacity corresponds to the hard-delay-constrained capacity established in our previous research [9], realized through a combination of diversity and channel inversion techniques. Note that several critical issues, including massive user access, the inter-dependency of data, and secure requirement provisioning, remain to be addressed concerning user-perceived capacity. In future research, we aim to expand this work to achieve immersive, highly dense connectivity, and secure wireless access technologies for emerging human-centric applications in 6G, such as extended reality, digital twins, and remote driving.

## Figures and Tables

**Table 1:** Running times of Algorithms 2 and 3.

*B*	50				100			
*T*	25	30	35	40	50	60	70	80
Algorithm 2	0.0106	0.0119	0.0120	0.0119	0.0331	0.0338	0.0462	0.0519
Algorithm 3	0.0033	0.00089	0.00091	0.0011	0.0036	0.0021	0.0030	0.0034

## Data Availability

Data are contained within this article.
